# A highlight on Sonic hedgehog pathway

**DOI:** 10.1186/s12964-018-0220-7

**Published:** 2018-03-20

**Authors:** Gabriela Basile Carballo, Jéssica Ribeiro Honorato, Giselle Pinto Farias de Lopes, Tania Cristina Leite de Sampaio e Spohr

**Affiliations:** 1 Laboratorio de Biomedicina do Cérebro, Instituto Estadual do Cérebro Paulo Niemeyer (IECPN), Secretaria de Estado de Saúde, Rua do Rezende 156, Centro, Rio de Janeiro, CEP: 20230–024 Brazil; 2 Laboratório de Hemato-Oncologia Celular e Molecular, Programa de Pesquisa em Hemato-Oncologia Molecular, Coordenação de Pesquisa, Instituto Nacional de Câncer (INCA), RJ, Brazil; 3 Programa de Pós-Gradução em Anatomia Patológica, Hospital Universitário Clementino Fraga Filho, Universidade Federal do Rio de Janeiro, Rio de Janeiro, Brazil

**Keywords:** Sonic hedgehog pathway, Canonical Shh signaling, Non-canonical Shh signaling, Clinical-trials, Brain tumors

## Abstract

Hedgehog (Hh) signaling pathway plays an essential role during vertebrate embryonic development and tumorigenesis. It is already known that Sonic hedgehog (Shh) pathway is important for the evolution of radio and chemo-resistance of several types of tumors. Most of the brain tumors are resistant to chemotherapeutic drugs, consequently, they have a poor prognosis. So, a better knowledge of the Shh pathway opens an opportunity for targeted therapies against brain tumors considering a multi-factorial molecular overview. Therefore, emerging studies are being conducted in order to find new inhibitors for Shh signaling pathway, which could be safely used in clinical trials. Shh can signal through a canonical and non-canonical way, and it also has important points of interaction with other pathways during brain tumorigenesis. So, a better knowledge of Shh signaling pathway opens an avenue of possibilities for the treatment of not only for brain tumors but also for other types of cancers. In this review, we will also highlight some clinical trials that use the Shh pathway as a target for treating brain cancer.

## Background

Hedgehog (Hh) is one of few of signaling pathways that is frequently used during development for intercellular communication. Hh is important for the organogenesis of almost all organs in mammals, as well as in regeneration and homeostasis. Further, Hh signaling is disrupted in diverse types of cancer [1, 2]. The vertebrate Hh signaling is not entirely dependent on an extremely specialized organelle, the primary cilium (PC), unlike other essential developmental signaling pathways. The PC is an organelle, microtubule-based, that emerges from the cell surface of most vertebrate cells. This organelle is important to process several cellular signals and/or extracellular environmental changes necessary for animal development, as Wingless (Wnt), Platelet-derived growth factor (PDGF), Shh, and Notch [3].

There are three mammalian Hh proteins, Shh, Indian-Hedgehog (Ihh), and Desert-Hedgehog (Dhh). Shh and Ihh have important, and sometimes coinciding, functions in several tissues. Shh has particularly marked roles in nervous system cell type specification and limbs patterning, whereas Ihh has important roles in skeletal development, mainly endochondral ossification. Dhh is restricted to the gonads including granulosa cells of ovaries and sertoli cells of testis [4–6]. The best-studied function of Shh, during mouse embryogenesis, is to instruct neural progenitors patterning, in which it is possible to distinguish six different cell types based on molecular markers, such as interneurons progenitors and motor neurons, that differentiate due to a gradient of Shh [7, 8].

Several evidences demonstrate that embryogenesis and tumorigenesis have common characteristics, where both processes depend on coordinated mechanisms of proliferation, differentiation and migration [9]. Vital signaling pathways for embryonic development and organogenesis are modulated in tumorigenesis. Aberrant activation of Hh signaling has been shown to be associated with the formation of brain tumors, as well as its cross talking with other pathways like transforming growth factor beta (TGFßs), Wnt, Notch and Shh [10–12]. Moreover, several studies have investigated the role of Hh-Gli (Gli means glioma-associated oncogene homologue) signaling in cancer initiating stem cells (CSCs) and suggested that it regulates self-renewal and tumorigenic potential [13]. This review focused on updating the role of these molecules in brain tumorigenesis as well as suggesting new therapeutic strategies/clinical trials using the Shh pathway as a potential future treatment.

### Shh signaling pathway components in tumorigenesis

#### The canonical pathway

**Fig. 1:**
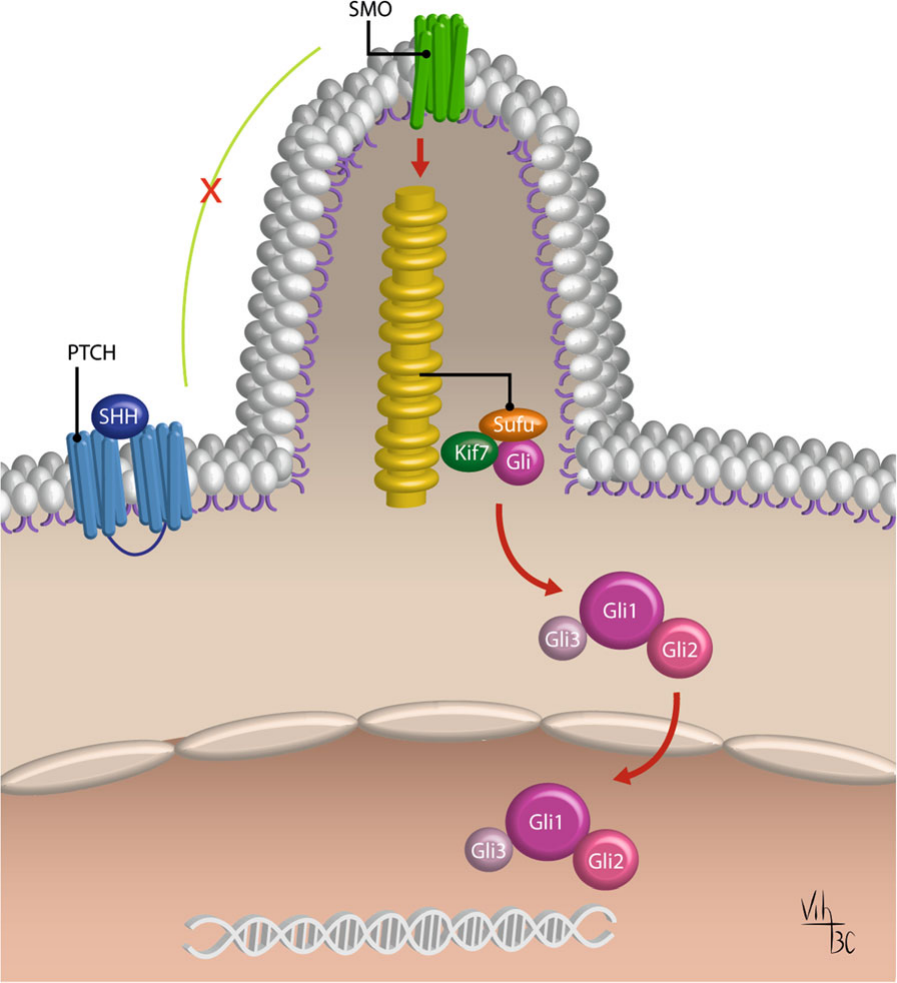
The Canonical activation of Shh pathway in vertebrates. The activation occurs by ligand-dependent interaction when Shh binds to Ptch at the cell membrane. In response to this binding, Ptch no longer inhibits Smo, which accumulates at the PC and initiates the downstream signaling pathway cascade. So, Smo regulates the Gli processing and activation at the PC. When Gli is activated, it translocates to the nucleus, where it activates target genes. (Diagram by Carballo, VC). (Adapted from Robbins et al., 2012) [54]

Activation of Shh pathway can happen in two major ways: 1. canonical signaling: by ligand-dependent interaction or through receptor-induced signaling and 2. non-canonical signaling, when there’s a mechanism of activation downstream of smoothened (Smo) (Fig. 1) [14].

The Shh canonical signaling occurs when the glycoprotein Shh binds and inactivates the 12-transmembrane protein Patched (Ptch1). In the lack of the ligand Shh, the activity of the 7-transmembrane protein Smo is inhibited by Ptch1, so Shh protein binding Ptch1 regulates Smo activity [15, 16]. Smo is a GPCR-like (G protein–coupled receptor) protein, and the translocation into the cilia membrane is a requisite for Gli activation [3, 17]. In response to Shh signaling, Ptch1 inhibition of Smo at the PC is abolished, when Ptch1 is internalized and degraded [18]. So, after Ptch1 degradation, Smo accumulates at the PC where is activated and stabilized by initiating the Shh downstream signaling cascade [18]. This downstream signaling cascade results in the translocation of Gli family proteins to the nucleus that begins the transcription of target genes, including Ptch1 and Gli1, in a negative and positive feedback loop, respectively (Fig. 1) [14]. Furthermore, Gli translocation to the nucleus also induces protein modulation of Wnt and Noggin [16, 19, 20]. Patched 2 (Ptch2) is another receptor for Shh that shares approximately 54% homology with Ptch1. However, the expression and signaling of Ptch2 is different from Ptch1, having decreased ability to inhibit Smo in absence of Shh ligand [21].

The Gli1 gene was initially cloned as an amplified oncogene of a malignant glioma and then characterized as a transcription factor of the hedgehog signaling pathway [22, 23]. Three Gli proteins (Gli1, Gli2 and Gli3) are zinc-finger transcription factors and are expressed in vertebrates, in overlapping and partially redundant domains. These three proteins are Shh-dependent, where only Gli1 occurs as a full-length transcriptional activator, while Gli2 and Gli3 act as either a negative or positive regulators (Gli2A - Gli2 activated or Gli2R - Gli2 repressor and Gli3A - Gli3 activated or Gli3R - Gli3 repressor, respectively) of the pathway which is determined by post-transcriptional and post-translational processing [24, 25]. Moreover, the change of Gli3A to Gli3R form is favored with respect to Gli2. Consequently, Gli2 has mainly an activator transcriptional behavior, while Gli3 acts as a repressor [26]. It has already been demonstrated that Gli2 can accumulate in the primary cilium and controls transcriptional activation, in response to Shh ligand binding, overcoming thereby the negative regulation of Gli3 [27].

The Gli3 has also a very important function in regulating Shh signaling. Without Shh, Gli3 has a repressor form (Gli3R). When Shh binds to Ptch and activates Smo, Smo converts Gli3R into an activated form (Gli3A). So, Gli3 works as a transcriptional factor with a dual function. The ratio of Gli3R/Gli3A is directly related to the control of several processes during organogenesis, such as digit types and number [28, 29].

**Fig. 2:**
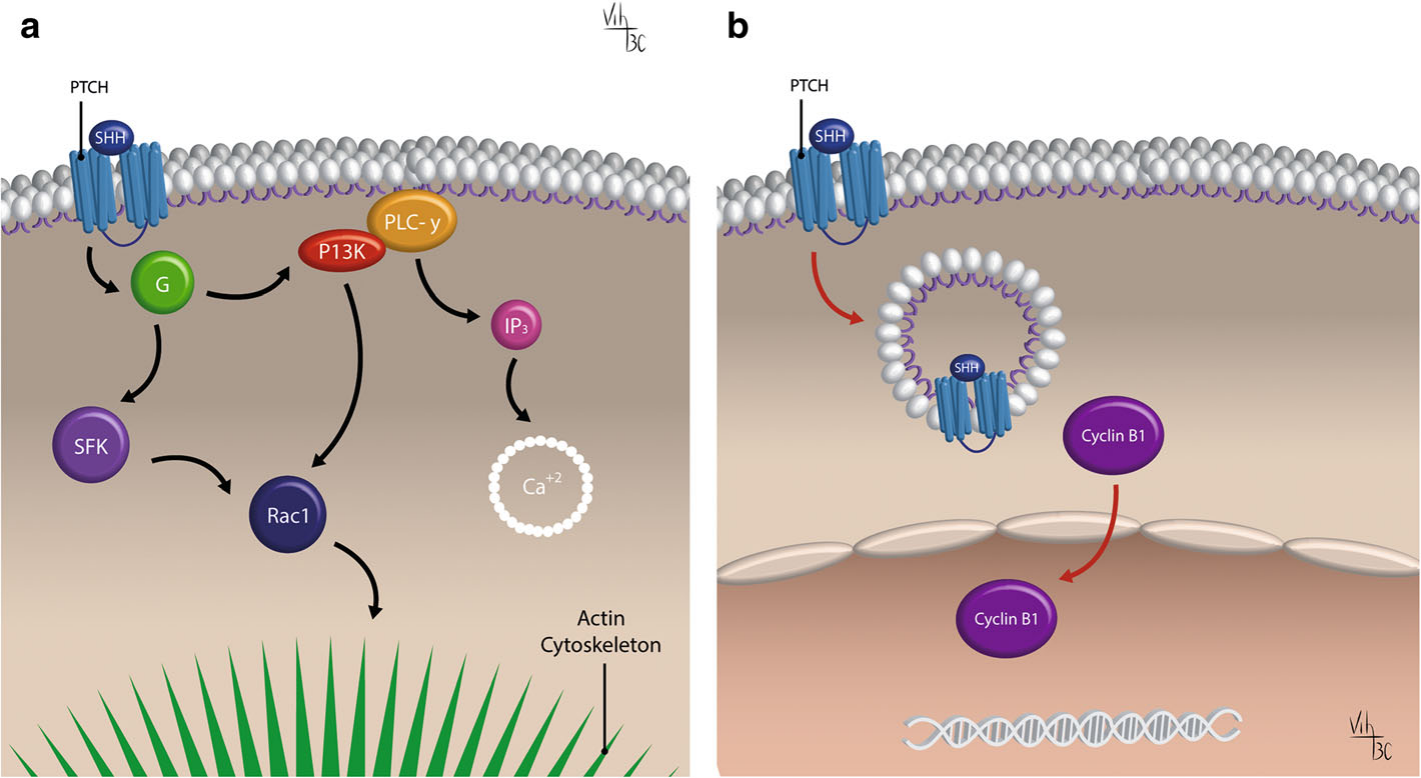
The non-canonical activation of Shh pathway. The non–canonical activation occurs through Gli-independent mechanisms and it can be of two types. A) Type I which modulates Ca^2+^ and actin cytoskeleton (left). When Shh binds the receptor Ptch, Smo is no longer inhibited and couple Gi proteins (G) and small GTPases RhoA and Rac1 activated. In addition, Smo stimulates calcium (Ca^2+^) release from the endoplasmic reticulum (ER) and PLC-γ-catalyzed the opening of IP3-dependent channels by the generation of IP3. B) Type II which is independent on Smo. When Shh binds Ptch, the interaction of Ptch with cyclin B1 is disrupted, leading to an increase in cell proliferation and survival (right). (Diagrams by Carballo, VC). (Adapted from Robbins et al., 2012) [54]

Shh signaling pathway can also be controlled by Supressor of Fused (SUFU) (Fig. 2) [30]. SUFU is a negative regulator of the Shh signaling pathway, acting on the Gli transcription factors. When Shh ligand is not present, SUFU binds directly the Gli proteins and inhibits their translocation to the nucleus, preventing the pathway activation [31]. However, the specific mechanisms concerning Gli inactivation by SUFU are not completely understood, but the full-length Gli proteins are converted to a C-terminal shorten repressor form: Gli-R. This truncated form of Glis is then partially degraded after subsequent phosphorylation by glycogen synthase kinase 3 beta (GSK3β), casein kinase I (CK1) and protein kinase A (PKA) [26]. Gli proteins retained at the cytoplasm by SUFU are then degraded or processed and thereby inhibiting Shh signaling [32]. When Gli-R moves to the nucleus, it represses SHH target genes including *Ptch1* and *Gli1* itself. When Shh pathway is activated, it is necessary that SUFU inhibition of Glis occurs by hyper-phosphorilation of SUFU [33]. Therefore, it has been previously demonstrated that several protein kinases, such as PKA and protein kinase C (PKC), CK1, mitogen activated protein kinase kinase (Mek1), GSK3, Phosphoinositide-3 kinase (PI3K), or dual specificity Yak1-related kinase (DYRK1) can modulate this pathway at several levels [33–39] (Fig. 2). This mechanism of regulation of the Shh pathway by ubiquitination-related posttranslational modifications of the Gli transcription factors leads to massive protein degradation or a proteasome-dependent proteolytic cleavage [40]. This process was first identified in *Drosophila*, but it was also demonstrated in vertebrates [41]. It is important to note that mutations in Ptch and SUFU, which are the negative regulators of Shh signaling, are linked to tumorigenesis, although the exact mechanism is unknown [42]. It was demonstrated in *knockout* mice, that the loss of SUFU is enough to activate the pathway without the support of the receptors [43, 44]. This constitutive Shh signaling activation in medulloblastoma (MB) is not sufficient to induce tumorigenesis, because a second tumor suppressor must be inactivated, such as p53 [45].

**Fig. 3:**
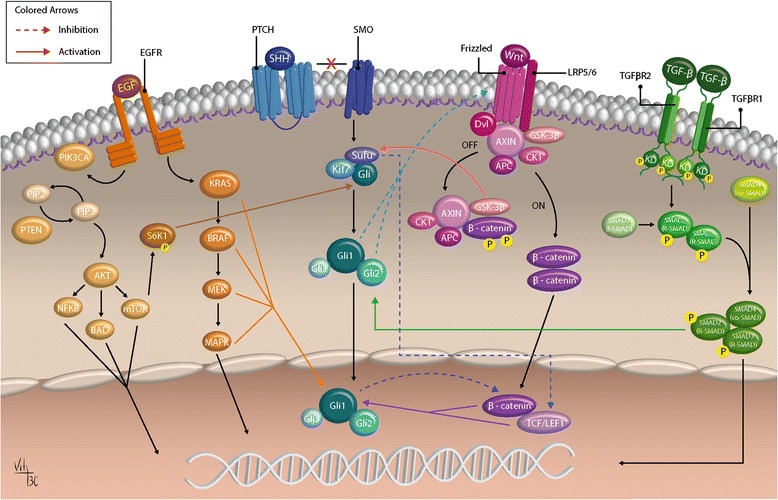
The crosstalk between Shh pathway and others. Shh signaling pathway can crosstalk with several pathways, especially EGF, Wnt and TGF-β. Here we can observe the Shh signaling pathway in blue, the EGF pathway in orange, the Wnt pathway in, and TGF-β pathway in green. The crosstalk between these pathways and Shh occurs at different moments, and it becomes more important to understand this molecular interaction in order to search for new therapeutical drugs. (Diagrams by Carballo, VC). (Adapted from Matias et al., 2017; Berg and Soreide, 2012 and https://www.mycancergenome.org/content/molecular-medicine/pathways/TGF-beta-signaling) [128, 129]

Besides ubiquitination, mainly of Gli3, to control Shh pathway, it was also demonstrated that Gli1 and Gli2 can be acetylated at lysine 518 and 757, respectively [46]. The mechanism of deacetylation of these proteins is mediated by the enzyme histone deacetylase 1 (HDAC1), which promotes transcriptional activation of the pathway. This activation is turned off by the degradation of HDAC1, which sustains a positive autoregulatory loop, when Shh is present. This degradation is mediated through an E3 ubiquitin ligase complex [46].

Shh signaling pathway is a valid therapeutic goal in a broad range of cancers, such as pancreas, prostate, breast and brain tumors. We focus here on brain tumors. The transcriptomics data on 149 clinical cases of The Cancer Genome Atlas-Glioblastoma (GBM) database showed a robust correlation between PTCH1 and GLI1 mRNA expression as an indication of the canonical Shh pathway activity in this malignancy. The expression of GLI1 mRNA varied in three orders of significance among the GBM patients of the same cohort, demonstrating a single continuous distribution different from the discrete high/low-GLI1 mRNA expressing clusters of MB [47]. Furthermore, it has already been well-established that tumor microenvironment plays an important role in controlling GBM pathology and their drug-resistance mechanisms [48]. Cells from the tumor microenvironment usually secrete inflammatory cytokines, growth factors [49–51] and other proteins that can activate Shh signaling in a typical or atypical manner (canonical or non-canonical) [52]. It was demonstrated that in the tumor microenvironment the endothelial cells provide Shh to activate the Hh signalling pathway in GBM cells, thereby promoting glioma stem cells (GSC) properties and tumor propagation [53].

#### Non-canonical Shh signaling

The “non-canonical Shh signaling” usually occurs through Gli-independent mechanisms. The Gli-independent mechanisms include two types: Type I is downstream of Smo, which modulates Ca^2+^ and actin cytoskeleton and type II is independent of Smo and increases cell proliferation and survival [54]. The non-canonical Shh signaling can regulate chemotaxis and cell migration through actin rearrangement. Additionally, it can stimulate cell proliferation via calcium-induced extracellular signal-regulated kinases (ERK) activation and activate Src family kinase, which is required axon guidance [54–56].

Some studies emerged mainly in tumor cells concerning the non-canonical Shh signaling in the ten last years. However it has not been completely elucidated how Smo selects between canonical or non-canonical routes. Usually the non-canonical route occurs when Smo couples to Gαi in vertebrates and modulates Ca^2+^ flux, Ras homolog gene family, member A (RhoA) and Rac activation and Warburg-like metabolism [56–58].

Interestingly, it was first believed that only Shh canonical signaling occurs when Smo enters the PC [59], and if Smo does not route through PC, it signals through a non-canonical pathway [17]. However, it was recently demonstrated that non-canonical Shh signaling leads to acetylation of α-tubulin via Smo-mediated calcium which increases in a primary cilia-dependent manner in mouse embryonic fibroblasts [17]. There are rare studies of this type of signaling associated with tumorigenesis and none with brain tumors. A ligand-independent Smo mutant resulted in tumors over-expressing Shh that show pronounced chromosomal instability and smoothened-independent up-regulation of Cyclin B1, a putative non-canonical branch of the Shh pathway in lung cancer. These results strongly support an autocrine, ligand-dependent model of Shh signaling in Small Cell Lung Cancer tumorigenesis and explain a new role for non-canonical Shh signaling through the induction of chromosomal instability [60]. Moreover, Hh signaling has an important role on the switch of hypoxia-induced pancreatic cancer epithelial to mesenchymal transition and invasion in a ligand-independent manner [61].

Recently, it was demonstrated that the intraflagellar transport protein 80 (IFT80) promotes Hh canonical signaling via activation of Hh-Smo-Ptch1-Gli signaling pathway during osteoblasts (OBs) differentiation. On the other hand, when this occurs, the non-canonical Hh signaling is inhibited via Hh-Smo-Gαi-RhoA-stress fibre signaling, demonstrating that non-canonical Hh signaling negatively regulates OBs differentiation [62]. Moreover, this study demonstrated that at least in OBs differentiation and bone formation, IFT80 is essential for the balance of the non-canonical and canonical Hh signaling pathways [62].

However, the researchers are still unveiling the mystery of the non-canonical Smo signaling axis, as well as how Smo selects between canonical and non-canonical routes.

#### Shh interaction with others pathways

It is already known that Shh signaling is very important for embryonic development and in adults, deregulation or mutation of this pathway plays an important role in both differentiation and proliferation, inducing tumorigenesis [63, 64]. Furthermore, CSCs follow the same pathways than normal stem cells such as Wnt, Shh, Notch and others and are also present during embryonic development, organogenesis and tumorigenesis [10–12].

Emerging evidence suggests that Shh signaling pathway can interact with other signaling components, such as TGF-β, epidermal growth factor receptor (EGFR), K-Ras, PKA, Notch, and Wnt/β-catenin (Fig. 3) [65, 66]. Furthermore, it has been suggested that more than one of these pathways are active, in different types of tumors, at the same time [16].

The Shh and Wnt pathways could interact in two ways: 1. through Gli1 and Gli2, which have been shown to regulate positively the expression of secreted frizzled-related protein-1 (sFRP-1) and thus inhibiting Wnt ligands and/or their receptors [67] and 2. through downstream GSK3β (an essential component of complexes that inhibit Shh and WNT morphogenetic pathways) [68]. GSK3β, can act as a positive regulator of Shh signaling by phosphorylating SUFU and promoting the release of SUFU from Gli, at least when the pathway is active [69]. It has already been demonstrated that in mice without normal APC function (citoplasmatic degradation and nuclear exporting of β-catenin) that SUFU negatively regulates Tcf-dependent transcription by reducing nuclear β-catenin levels [70]. So, Shh can regulate Wnt signaling. This crosstalk between Shh and Wnt has also been demonstrated in medulloblastoma cells, where the loss of SUFU activates both pathways, inducing excessive proliferation and tumorigenesis [71]. Besides, Wnt signaling can also increase Shh pathway activity, as β-catenin may potentially affect the Gli1 transcriptional activity via TCF/LEF in an independent manner [66]. Interestingly, in gastric cancer, the Shh signaling pathway activation seems to be inversely correlated with the level of Wnt pathway activation. It was observed that Gli1 overexpression suppressed Wnt transcriptional activity, nuclear β-catenin accumulation and proliferation of gastric cancer cells [72].

It is well established that aberrant RAS activation has a protagonist role in tumorigenesis, and activating RAS mutation occurs in 30% of all human cancers [73]. It has been demonstrated that activation of the RAS/MAPK pathway (KRAS), induced by divers upstream signals and converging at the level of Gli transcription factors, is important in promoting cancer development during pancreatic tumorigenesis [74]. Another pathway that has been demonstrated to interact with Shh is the ERK signaling pathway, which controls Gli transcription factor function in Shh signaling, when stimulated by exogenous ligands (like basic fibroblast growth factor -bFGF) [39].

In addition to Wnt/βcatenin and KRAS, TGF-β/TGF-βR, EGFR, and platelet-derived growth factor receptor α (PDGFRα) can also cooperate with the canonical Shh pathway [39, 66, 75–78]. There is an important increase in Gli1 and Gli2 expression induced through the activation of TGF-β/TGF-βR/Smads in pancreatic cell lines. Furthermore, these cells were resistant to Shh inhibition, but the pharmacologic blockade of TGF-β signaling leads to repression of cell proliferation accompanied by a reduction in Gli2 expression [79].

Another signaling pathway that crosstalk with Shh and contributes to tumorigenesis is EGFR signaling. The stimulation of EGFR/RAS/RAF/MEK/ERK in different cancer cell lines such as gastric, and pancreatic cancer cell lines and was able to activate the Gli transcription factor and selective transcriptional modulation of Gli target gene expression [76–78, 80]. However, it was observed in MB cells, a crosstalk mechanism where EGFR signaling silences proteins acting as negative regulators of Hh signaling, as an ERK and AKT-signaling independent method. Reciprocally, a high-level synergism was also observed, due to a significant and strong up-regulation of several canonical EGF-targets. Synergistic outcomes between EGFR and Hh signaling can selectively promote a shift from a canonical HH/GLI profile to a gene profile specific target modulated. It indicates that there are more diffuse, yet context-dependent (i.e. cancer-dependent) interactions, between growth factor receptors and HH/GLI signaling in human tumorigenesis [81].

So, it is becoming more and more evident that the integration of these signaling pathways, which are important for embryonic morphogenesis, may support a more malignant behavior by tumor cells and consequently maintain the tumorigenesis of diverse aggressive tumors, such as pancreas, prostate, breast and brain [78, 80]. The need to understand the role of these pathways in tumorigenesis is becoming increasingly evident, mainly the molecular crosstalk between them, as it is an important consideration for the development of HH-targeting agents and the appropriate selection of a class of inhibitors for therapeutic intervention [82]. Furthermore, it is valid to be proposed in the future treatments of Shh-dependent tumors using inhibitors of Akt, PI3K, MEK, ERK, Wnt, EGFR and TGF-β [38, 66, 78–80].

### Hh inhibitors and clinical trials

#### The importance of stem cells in brain tumors

Nowadays, several studies support the hypothesis that malignant tumors are initiated and maintained by CSCs. Although the origin of the CSCs in human tumors is not fully understood, it is already well established that these cells are responsible for the chemo and radioresistance of the most malignant tumors [49, 50]. The recurrence of the tumor is usually due the existence of these cells in the tumor bulk [49, 50]. Moreover, studies have demonstrated that CSCs could de-differentiate from a more differentiated cancer cell present in the tumor mass that acquires self-renewal properties, clonal tumor initiation capacity and clonal long-term repopulation potential, perhaps as a result of epithelial-to-mesenchymal transition (EMT) [83–85].

The hypothesis that the existence of CSCs initiates malignant tumor came from the observation that tumor cells, like adult tissues, originate from cells that can self-renew. Furthermore, that these cells also are able to differentiate into cell forming the tumor bulk [86]. In the adult tissue, these cells are the adult stem cells that are tissue-specific and multipotent, being able to differentiate between all cell types of the tissue of origin [86].

In the adult brain, it is already well established that the existence of a neurogenic niche, which is extremely dynamic and complex microenvironment where new glial cells and neurons are generated when necessary from the stem or progenitor cells [87]. This neurogenic niche has a very important role, as it provides signals that regulate whether the stem cells should differentiate, remain quiescent, or actively divide, controlling the self-renewal properties in this way and maintaining neural stem/progenitor cell populations [87]. These neural stem cells (NSCs) are found in two main niches in adult brain, in the lateral ventricles (ventricular-subventricular zone (V-SVZ)) and in the hippocampus (subgranular zone (SGZ)), and these microenvironments ensures the self-renew and multipotent properties [88, 89].

It is interesting to note that Shh is very important for determining cell fate and patterning during embryo development, having a mitogenic effect on proliferative cells throughout development [90]. Recently it was demonstrated that in the adulthood, the level of Shh signaling pathway activation played an important role to regulate the balance between quiescent and activated NSCs. Moreover, when the Shh pathway was genetically activated the number of quiescent NSCs increased and the pool of activated NSCs decreased [91]. However, there was an initial transitory period over the short term when activated NSCs are actively proliferating, apparently when their G1 and S-G2/M phases were short [91].

Taking into account that in GBM, the Shh pathway is usually upregulated, affecting GBM CSC proliferation and self-renewal [87, 92], this discovery opened an avenue for clinical trials that managed not only to stop the tumor to growth but also the tumor to relapse after surgery.

#### The importance of Shh and MGMT interaction in clinical trials

Nowadays, the standard treatment for most brain tumors comprises resection of the majority of the tumor mass, followed by chemo- and radiotherapy [49, 93], being Temozolmide (TMZ) and radiotherapy being the gold standard treatment [94]. TMZ is an alkylating agent prodrug, and its effect on tumor cells is to methylate the O6 residues of guanine preventing DNA duplication during cell proliferation and inducing cell death and apoptosis [95]. However, the DNA repair enzyme O-6-methylguanine-DNA methyltransferase (MGMT) is able to reverse the effects of alkylating agents as TMZ [96–98]. The MGMT promoter methylation is directly related to patient’s prognosis, as low promoter methylation status induces a high MGMT expression and a shorter survival due to a remarkable chemoresistance. On the other hand, a higher promoter methylation status predicts a good response to TMZ chemotherapy, as the MGMT enzyme is downregulated, resulting in longer survival for the patients [99, 100]. Therefore, studies are being done in order to control and impairs the MGMT enzyme activity in chemoresitant tumors It is interestingly to note that many DNA repair proteins could be potential targets for inhibiting cancer cells without affecting normal cells; as they usually are upregulated in several chemorresistant cells and cancers [101].

The most malignant tumors are also highly mutated and present CSCs, which make them difficult to treat. So efforts are being made in order to bypass the chemoresitance in tumors. As written above, the Shh pathway is upregulated in CSCs [87, 102]. Moreover, these cells express also usually high levels of MGMT, and therefore they are involved in chemotherapy resistance and are responsible for tumor recurrence [103]. Emerging evidences are demonstrating that Shh signaling pathway could regulate MGMT expression and chemoresistance to TMZ in human GBM. Moreover, this regulation occurred independently from MGMT promoter methylation status, offering a probable target to reestablish chemosensitivity to TMZ in tumor that developed chemoresitance [104]. Furthermore, it is believed that Gli1 expression is also responsible for chemoresistance in gliomas and that it’s overexpression is related to tumor recurrence after treatment. So in the other hand, when Shh pathway is inhibited, [105] the sensitivity to chemotherapy improves by down-regulating many genes related to apoptosis, cell survival, multi-drug resistance, and especially MGMT [102, 106–109].

#### Smo-based inhibitors

Presently, there are several Hh inhibitors employed in clinical trials for different types of brain tumors (www.clinicaltrials.gov) (see Table 1). SMO is the principal target for the development of Shh-pathway inhibitors; however preclinical and clinical studies have demonstrated that the use of Smo inhibitors induces the development of mutations that lead to treatment resistance [110, 111].

**Table 1:** Hedegehog Pathway Inhibitors

	Compound	Where it acts
Biological-based inhibitors	3H8, 6D7 (antibody)	Shh pathway inhibitor
	Cyclopamine	Smo inhibitor
	5E1 Antibody	Shh pathway inhibitor
	Isoflavon (Genistein)	Shh pathway inhibitor
	Curcumin	Gli 1 inhibitor
	Resveratrol	Gli 1 inhibitor
	Epigallocatechin-3-gallate	Gli 1 inhibitor
	Physalin B and Physalin F	Gli 1 inhibitor
	Jervine	Smo inhibitor
	Zerumbone	Gli 1 inhibitor
	Staurosporinone	Gli 1 inhibitor
	Vitamin D3	Smo inhibitor
Chemical Based	GDC-0449 (Vi sm odegib/Erivedge™)	Smo inhibitor
	IPI-926 (Saridegib)	Smo inhibitor
	NVP-LDE225 (Erismodegib) (Sonidegib)	Smo inhibitor
	PF-04449913 (Glasdegib)	Smo inhibitor
	BRD-6851	Smo inhibitor
	LY2940680	Smo inhibitor
	MK-5710	Smo inhibitor
	SEN450	Smo inhibitor
	PF-5274857 (A-116)	Smo inhibitor
	MRT-10 and MRT-14	Smo inhibitor
	TAK-441	Smo inhibitor
	SANT1, SANT2, SANT3, SANT4, SANT74 and SANT75	Smo inhibitor
	MS-0022	Smo inhibitor
	Arsenic Trioxide (ATO)	Gli 1 inhibitor
	Sodium Arsenite	Gli 1 inhibitor
	HPI-1, HPI-2, HPI-3 and HPI-4	Gli inhibitors
	AKI0532	Probably Smo inhibitor
	Itraconazole	Smo inhibitor
	GANT 58, GANT 61	Gli 1 inhibitor
	KAAD-Cyclopamine	Smo inhibitor
	Cur-61,414	Smo inhibitor
	Robotnikinin	Shh pathway inhibitor
	SAG	Smo inhibitor
	Purmorphamine	Smo inhibitor
	BMS-833923 (XL139)	Smo inhibitor
	LY2940680 (Taladegib)	Smo inhibitor
	MRT-92	Smo inhibitor
	PF-5274857	Smo inhibitor
	LEQ506	Smo inhibitor
	RU-SKI 43	Shh pathway inhibitor
	Imiquimod	Shh pathway inhibitor
	Patidegib	Shh pathway inhibitor

The first clinical trial, targeting Smo and so using Shh pathway inhibitor as therapy, considered several patients with recurrent or metastatic basal cell carcinoma (BCC). At that time, a preliminary study was performed with cyclopamine in a topical application and cream formulation. This study has revealed that the tumors rapidly regressed in all cases without adverse effects, and the normal skin and putative stem cells exposed to cyclopamine were preserved [112]. Cyclopamine is a natural steroidal alkaloid derived from *Veratrum californicum* which inhibits the cellular response to Shh signaling by antagonizing the proto-oncogene SMO [113]. The histological and immunohistochemical analyses from this study have also indicated that the topical cyclopamine application resulted in an inhibition of the proliferation and induced the apoptotic death of tumor cells [112]. In 2006, Herman started a Phase III clinical trial to assess cyclopamine as a chemo-preventive agent to inhibit the recurrence of BCC following surgical resection. At that moment, neither a phase I nor a phase II clinical trials have evaluated the possible side effects of cyclopamine in human subjects, so the patients may choose not to take part in the study. It is important to note that in both clinical trials, the cyclopamine was administered topically that diminished the side effects of the drug [112, 114].

However, cyclopamine has never been used orally in clinical trials. Test using animal models demonstrated that cyclopamine besides being poorly soluble orally, at high doses, it has a potential teratogenic effect, causing many potential side effects, including weight loss, dehydration, and death [115], which limits its clinical use. Therefore, some other potent SMO inhibitors have also reached the clinical trials, such as: the orally active IPI-926, a semi-synthetic derivative of cyclopamine and different synthetic compounds, such as GDC-0449 (vismodegib), Cur61414, and NVPLDE-225 (Erismodegib or Sonidegib or Odomzo) [116–119].

Presently some ongoing and completed clinical trials used Shh inhibitors to treat brain tumors (see Tables 2 and 3). The first clinical trial performed using a Shh inhibitor to treat a brain tumor was conducted in 2008. At the time, a 26-year-old man with metastatic MB that was refractory to multiple therapies was treated with a novel Hh pathway inhibitor, GDC-0449 [117]. Interestingly, the group did molecular analyses of the patient’s tumor specimens obtained before treatment which suggested activation of Shh pathway, as there was a high expression of Hh target genes including GLI1, PTCH1, PTCH2 and sFRP1. So, the treatment resulted in rapid regression of the tumor and reduction of symptoms, but unfortunately, this effect was transient and the patient died after five months of treatment [117]. It was observed that the Hh pathway inhibition with GDC-0449 induced the malignant transformation in MB which induced the tumor regrowth and the rapid progress of the disease [117].

**Table 2:** Ongoing Clinical Trials

Study	ClinicalTrials .gov Identifier	Sponsor	Tumor	Phase	Shh Drug inhibitor	Where it acts
Arsenic Trioxide, Temozolomide, and Radiation Therapy in Treating Patients With Malignant Glioma That Has Been Removed By Surgery	NCT00275067	Northwestern University Collaborators: Cephalon CTI BioPharma	Brain and Central Nervous System Tumors	Phase 1 Phase 2	arsenic trioxide	Gli 1 inhibitor
Vismodegib and FAK Inhibitor GSK2256098 in Treating Patients With Progressive Meningiomas	NCT02523014	Alliance for Clinical Trials in Oncology Collaborators: National Cancer Institute (NCI) GlaxoSmithKline Genentech, Inc. Brain Science Foundation	Intracranial Meningioma Recurrent Meningioma	Phase 2	GDC-0449 (vismodegib) (Erivedge)	Smo inhibitor
A Clinical and Molecular Risk-Directed Therapy for Newly Diagnosed Medulloblastoma	NCT01878617	St. Jude Children’s Research Hospital Collaborators: Genentech, Inc. National Cancer Institute (NCI)	Medulloblastoma	Phase 2	GDC-0449 (vismodegib) (Erivedge)	Smo inhibitor
Study of Vismodegib in Combination With Temozolomide Versus Temozolomide Alone in Patients With Medulloblastomas With an Activation of the Sonic Hedgehog Pathway	NCT01601184	Centre Leon Berard Collaborator: Ministry of Health, France	Medulloblastoma	Phase 1 Phase 2	GDC-0449 (vismodegib) (Erivedge)	Smo inhibitor
NCT Neuro Master Match - N^2^M^2^ (NOA-20) (N^2^M^2^)	NCT03158389	University Hospital Heidelberg Collaborators: German Cancer Aid German Cancer Research Center National Center for Tumor Diseases, Heidelberg	Adult Glioblastoma	Phase 1 Phase 2	GDC-0449 (vismodegib) (Erivedge)	Smo inhibitor
Study of Genistein in Pediatric Oncology Patients (UVA-Gen001) (UVA-Gen001)	NCT02624388	University of Virginia	Neuroblastoma, Rhabdomyosarcoma, Medulloblastoma, Brain Neoplasms	Phase 2	Genistein	Gli 1 inhibitor
A Proof-of-concept Clinical Trial Assessing the Safety of the Coordinated Undermining of Survival Paths by 9 Repurposed Drugs Combined With Metronomic Temozolomide (CUSP9v3 Treatment Protocol) for Recurrent Glioblastoma	NCT02770378	University of Ulm Collaborators: Reliable Cancer Therapies Anticancer Fund, Belgium	Glioblastoma	Phase 1	itraconazole	Smo inhibitor

**Table 3:** Complete Clinical Trials

Study	ClinicalTrials .gov Identifier	Sponsor	Tumor	Phase	Shh Drug inhibitor	Where it acts	Outcome of the clinical trials
Arsenic Trioxide in Treating Patients With Advanced Neuroblastoma or Other Childhood Solid Tumors	NCT00024258	Memorial Sloan Kettering Cancer Center Collaborator: National Cancer Institute (NCI)	Brain and Central Nervous System Tumors	Phase 2	arsenic trioxide	Gli 1 inhibitor	Limitations of the study, such as early termination leading to small numbers of participants analyzed and technical problems with measurement leading to unreliable or uninterpretable data
Radiation Therapy, Arsenic Trioxide, and Temozolomide in Treating Patients With Newly Diagnosed High-Grade Glioma	NCT00720564	City of Hope Medical Center Collaborator: National Cancer Institute (NCI)	Brain and Central Nervous System Tumors	Phase 1	arsenic trioxide	Gli 1 inhibitor	No Study Results Posted
Arsenic Trioxide and Radiation Therapy in Treating Young Patients With Newly Diagnosed Gliomas	NCT00095771	Sidney Kimmel Comprehensive Cancer Center Collaborator: National Cancer Institute (NCI)	Brain and Central Nervous System Tumors	Phase 1	arsenic trioxide	Gli 1 inhibitor	No Study Results Posted
Phase I Trial of Arsenic Trioxide and Stereotactic Radiotherapy for Recurrent Malignant Glioma	NCT00185861	Stanford University Collaborators: Cephalon CTI BioPharma	Brain Cancer	Phase 1	arsenic trioxide	Gli 1 inhibitor	No Study Results Posted
Arsenic Trioxide Plus Radiation Therapy in Treating Patients With Newly Diagnosed Malignant Glioma	NCT00045565	National Cancer Institute (NCI)	Adult Giant Cell Glioblastoma Adult Glioblastoma Adult Gliosarcoma	Phase 1	arsenic trioxide	Gli 1 inhibitor	No Study Results Posted
Curcumin Bioavailability in Glioblastoma Patients	NCT01712542	Johann Wolfgang Goethe University Hospital	Glioblastoma	Early Phase 1	Curcumin	Shh inhibitor	No Study Results Posted
GDC-0449 in Treating Young Patients With Medulloblastoma That is Recurrent or Did Not Respond to Previous Treatment	NCT00822458	National Cancer Institute (NCI)	Recurrent Childhood Medulloblastoma	Phase 1	GDC-0449 (vismodegib) (Erivedge)	Smo inhibitor	No Study Results Posted
GDC-0449 in Treating Patients With Recurrent Glioblastoma Multiforme That Can Be Removed by Surgery	NCT00980343	National Cancer Institute (NCI)	Adult Giant Cell Glioblastoma Adult Glioblastoma Adult Gliosarcoma Recurrent Adult Brain Tumor	Phase 2	GDC-0449 (vismodegib) (Erivedge)	Smo inhibitor	The only disclosure restriction on the PI is that the sponsor can review results communications prior to public release and can embargo communications regarding trial results for a period that is less than or equal to 60 days. The sponsor cannot require changes to the communication and cannot extend the embargo.
Vismodegib in Treating Younger Patients With Recurrent or Refractory Medulloblastoma	NCT01239316	National Cancer Institute (NCI)	Recurrent Childhood Medulloblastoma	Phase 2	GDC-0449 (vismodegib) (Erivedge)	Smo inhibitor	Resulted in the following paper:
							Robinson et al., 2015
Vismodegib in Treating Patients With Recurrent or Refractory Medulloblastoma	NCT00939484	National Cancer Institute (NCI)	Adult Medulloblastoma	Phase 2	GDC-0449(vismodegib) (Erivedge)	Smo inhibitor	Resulted in the following paper: Robinson et al., 2015
Erivedge (Vismodegib) in the Treatment of Pediatric Patients With Refractory Pontine Glioma	NCT01774253	Giselle Sholler Collaborators: Spectrum Health Hospitals Phoenix Children’s Hospital	Pontine Glioma	Phase 2	GDC-0449(vismodegib) (Erivedge)	Smo inhibitor	Limitations of the study, such as early termination leading to small numbers of participants analyzed and technical problems with measurement leading to unreliable or uninterpretable data Posted
Efficacy of Prophylactic Itraconazole in High-Dose Chemotherapy and Autologous Hematopoietic Stem Cell Transplantation	NCT00336531	Samsung Medical Center	Neuroblastoma Brain Tumor Retinoblastoma	Phase 4	itraconazole	downregulatio n in GLI	No Study Results Posted
A Dose Finding and Safety Study of Oral LEQ506 in Patients With Advanced Solid Tumors	NCT01106508	Novartis Pharmaceuticals	Recurrent or Refractory Medulloblastoma	Phase 1	LEQ506	Smo inhibitor	No Study Results Posted
Dose Finding and Safety of Oral LDE225 in Patients With Advanced Solid Tumors	NCT00880308	Novartis Pharmaceuticals	Medulloblastoma	Phase 1	LDE225(Sonidegib)	Smo inhibitor	No Study Results Posted
An East Asian Study of	NCT01208831	Novartis Pharmaceuticals	Medulloblastoma	Phase 1	LDE225	Smo inhibitor	No Study Results
LDE225					(Sonidegib)		Posted
A Phase I Dose Finding and Safety Study of Oral LDE225 in Children and a Phase II Portion to Assess Preliminary Efficacy in Recurrent or Refractory MB	NCT01125800	Novartis Pharmaceuticals	Medulloblastoma Rhabdomyosarcoma Neuroblastoma Hepatoblastoma Glioma Astrocytoma	Phase 1 Phase 2	LDE225 (Sonidegib)	Smo inhibitor	Other disclosure agreement that restricts the right of the PI to discuss or publish trial results after the trial is completed.
Phase Ib, Dose Escalation Study of Oral LDE225 in Combination With BKM120 in Patients With Advanced Solid Tumors	NCT01576666	Novartis Pharmaceuticals	Recurrent Glioblastoma Multiforme	Phase 1	LDE225 (Sonidegib)	Smo inhibitor	No Study Results Posted
A Phase II Study of Oral LDE225 in Patients With Hedge-Hog (Hh)- Pathway Activated Relapsed Medulloblastoma (MB)	NCT01708174	Novartis Pharmaceuticals	Medulloblastoma	Phase 2	LDE225 (Sonidegib)	Smo inhibitor	Other disclosure agreement that restricts the right of the PI to discuss or publish trial results after the trial is completed
Phase 1 Multiple Ascending Dose Study of BMS-833923 (XL139) in Subjects With Solid Tumors	NCT01413906	Bristol-Myers Squibb	Solid Tumors including Glioblastoma	Phase 1	BMS- 833923 (XL139)	Smo inhibitor	No Study Results Posted
Imiquimod/Brain Tumor Initiating Cell (BTIC) Vaccine in Brain Stem Glioma	NCT01400672	MasonicCancer Center, Universityof Minnesota	Diffuse Intrinsic Pontine Glioma	Phase 1	Imiquimod	Shhpathwayi nhibitor	No Study Results Posted

Only in 2012, the US Food and Drug Administration (FDA) approved GDC-0449 as a standard therapy in patients with locally advanced and metastatic BCC [120]. Then few phase I and II clinical trials emerged with the objective to define the pediatric maximum tolerated dose and the efficacy of GDC-0449 in SHH-MB. Some studies evaluated the use of GDC-0449 in combination with TMZ. The studies were performed through the Pediatric Brain Tumor Consortium. These were phase II studies which evaluated the efficacy of GDC-0449 in younger patients, as well as in adult patients with recurrent or refractory MB [121]. Many other collaborative studies using GDC-0449 are still ongoing which directs therapy based on both clinical and molecular risk stratification (see Table 2 and see www.clinicaltrials.gov). GDC-0449 has an advantage for the use in clinical trials since it has low toxicity and high specificity for the Shh pathway. Additionally, this drug may also be used together with other pathway inhibitors or chemotherapy [122]. Moreover, GDC-0449 usually is well tolerated because of a lack of Smo receptor in most normal tissues [111]. It is believed that the use of Shh pathway inhibitors in MB treatment may offer an adequate therapeutic option. However, it is important to note that, as Shh pathway is very important during development, the adverse effect of blocking Shh pathway in prepubescent children is not completely understood [123]. Recently a study demonstrated that the used of GDC-0449 in pediatric oncologic patients induces short stature and growth abnormalities as they developed physeal fusion [124]. So, the use of Hh inhibitors in skeletally immature patients should be widely discussed and may be limited to those patients whom treatment options are limited or absent.

#### Gli-based inhibitors

Shh-MBs as GBMs are highly mutated tumors, and it is not uncommon for those tumors to demonstrate primary resistance to SMO inhibition, as they present alterations in downstream SHH pathway genes such as SUFU, GLI2, or MYCN [125]. As described above, it is typical for those tumors to acquire secondary resistance to Shh inhibition, and in this case, a Shh inhibition monotherapy is not efficient [117]. This is why, several pharmaceutical companies such as Exelixis/Bristol-Myers Squibb, Novartis, Infinity, and Pfizer developed alternative Shh antagonists that act directly in Gli (see Table 1). Some of these inhibitors are already being tested in the brain and central nervous system tumors as coadjuvant therapy with TMZ (see Tables 2 and 3 and www.clinicaltrials.gov).

So, besides GDC-0449, NVPLDE-225 and BMS-833923 (XL139) were also tested in brain tumors. There were some phase I and phase II clinical trials completed with the purpose of testing the efficacy tolerability, pharmacokinetics, pharmaco-dynamics, and safety of these drugs orally [65].

Another drug that is being tested for gliomas in phase I and II clinical trials is the arsenic trioxide (ATO). ATO is an FDA-approved drug that has been shown to inhibit Gli-dependent growth in MB mouse model, which was first used for the treatment of patients with acute promyelocytic leukemia (APL) [126]. Recently, a study demonstrated that apparently, the treatment of patients in combination with ATO, TMZ, and radiation does not improve the overall outcome in GBM patients; however, it might have some benefit in anaplastic astrocytoma patients [127].

Most of Hh inhibitors that have entered clinical trials targeted Smo, although several mechanisms of resistance to Smo inhibitors have been identified. Therefore, the discovery of new Hh pathway inhibitors may be crucial to bypass these resistance mechanisms and control tumorigenesis.

## Conclusions

The Shh pathway is a well-established pillar of neural development and cancer cells use this mechanism to resist therapy and recur. The Shh pathway is thought to be very simple, as it usually signals canonically through Gil proteins; however, the shh pathway can be very complex, as demonstrated by the emerging evidence. Moreover, this pathway can not only be controlled through several mechanisms and molecules, such as Gli2R and Gli3R, SUFU and Ptch, which are components of the pathway, but also through posttranslational modifications, such as ubiquitination and acetylation. Several reports demonstrated that Shh could also signal through a non-canonical route; however, it is still a mystery how the cells select between canonical and non-canonical routes. Shh pathway can also interact with other signaling components that are important during embryonic development and tumorigenesis, such as TGF-β, EGFR, and Wnt. Cross-talking between these pathways and Shh signaling plays a pivotal role in the presevation of CSCs postulated to have intrinsic resistance to chemotherapy. So, a better understanding of the mechanisms is involved in the interaction between Shh pathway, and these pathways open a huge window of opportunities for the development of new therapeutic drugs for multiple cancers. Moreover, the inhibition of Shh signaling components may prove to be key to resistance and potential therapeutic targets to GBM and MB. The CSC hypothesis provides an explanation for the heterogeneity and recurrence of these tumors, and the Shh signaling pathway plays an important role in the maintenance of these cells. However, we believe that the best way to control the turmor recurrence is combining Shh antagonist with convetional therapies that are actually used in the clinic. Nowadays, the primary target used for development of Shh-pathway inhibitors in clinical trials is SMO, and there are several clinical trials for different types of brain tumors ongoing. So, current clinical trials offer a great outlook to overcome brain tumor. But, we still believe that more researchers must be conducted, as unfortunately we did not reach the cure for most of the cancers, such as GBMs and MBs, that are very aggressive.

## Abbreviations

APL: Promyelocytic leukemia
ATO: Arsenic trioxide
BCC: Basal cell carcinoma
bFGF: Basic fibroblast growth factor
CK1: Casein kinase 1
CSCs: Cancer initiating stem cells
Dhh: Desert-Hedgehog
DYRK1: Dual specificity Yak1-related kinase
EGFR: Epidermal growth factor receptor
EMT: Epithelial-to-mesenchymal transition
ERK: Extracellular signal-regulated kinases
FDA: Food and Drug Administration
GBM: Glioblastoma
GDC-0449: Vismodegib
Gli: Glioma-associated oncogene homologue
Gli2A: Gli2 activated
Gli2R: Gli2 repressor
Gli3A: Gli3 activated
Gli3R: Gli3 repressor
GPCR: G protein–coupled receptor
GSC: Glioma stem cells
GSK3: Glycogen synthase kinase 3
HDAC1: Histone deacetylase 1
Hh: Hedgehog
IFT80: Intraflagellar transport protein 8
Ihh: Indian-Hedgehog
MB: Medulloblastoma
Mek1: Mitogen activated protein kinase kinase
MGMT: O-6-methylguanine-DNA methyltransferase
NSCs: Neural stem cells
NVPLDE-225: Erismodegib or Sonidegib or Odomzo
Obs: Osteoblasts
PC: primary cilium
PDGF: Platelet-derived growth factor
PDGFRα: Platelet-derived growth factor receptor α
PI3K: Phosphoinositide-3 kinase
PKA: Protein kinase A
PKC: Protein kinase C
Ptch1 and 2: Patched 1 and Patched 2
RhoA: Ras homolog gene family, member A
sFRP-1: Secreted frizzled-related protein-1
SGZ: Subgranular zone
Shh: Sonic-Hedgehog
Smo: Smoothened
SUFU: Supressor of Fused
TGFß: Transforming growth factor beta
TMZ: Temozolamide
V-SVZ: Ventricular-subventricular zone
Wnt: Wingless
